# Instruments for Assessing Health-Related Quality of Life in People With Visual Impairment: Protocol for a Scoping Review

**DOI:** 10.2196/81976

**Published:** 2026-05-13

**Authors:** Aline Fernanda Alves Ribeiro, Marcos Antonio Ferreira Júnior, Aline Moraes da Silva, Leticia Lima Meza, Bethânia Karoline Alvaro Menezes

**Affiliations:** 1 Postgraduate Program in Nursing, Integrated Health Institute, Federal University of Mato Grosso do Sul, Costa e Silva Avenue, Pioneiros neighborhood, Campo Grande, 79070-900, Brazil, 55 (67) 99850-6070

**Keywords:** quality of life, instruments, people with visual impairment, assessment, scoping review, nursing

## Abstract

**Background:**

Visual impairment affects approximately 2.2 billion people worldwide and has significant impacts on various aspects of life, including physical, social, economic, and emotional domains. Assessing the quality of life of these individuals is essential for identifying their needs and guiding health promotion strategies. However, no studies were found that systematically cataloged the instruments used for this evaluation specifically for people with visual impairment.

**Objective:**

This study aims to systematically map the scientific evidence regarding the instruments used to assess quality of life in individuals with visual impairment at any health care level.

**Methods:**

The population, concept, and context framework guided the development of the research question: What instruments are available in the scientific literature to assess the quality of life of people with visual impairment across health care levels? Data will be collected from major databases and gray literature, with duplicates managed in Mendeley and screening conducted independently by 2 reviewers using Rayyan. Full texts will be assessed based on eligibility criteria, and data will be synthesized in Microsoft Excel and reported using a flowchart and narrative summary, following PRISMA-ScR (Preferred Reporting Items for Systematic Reviews and Meta-Analyses Extension for Scoping Reviews) guidelines.

**Results:**

This protocol was registered on the Open Science Framework platform on July 28, 2025. The results of this study will be disseminated through publication in a peer-reviewed scientific journal. It is expected that the findings will provide valuable support for the development and advancement of a broader research project.

**Conclusions:**

Identifying and evaluating instruments used to assess the quality of life in individuals with visual impairment are crucial to ensure the use of reliable and scientifically sound tools. This process not only advances scientific knowledge but also informs public health policies aimed at promoting equity, inclusion, and improved living conditions for this population.

## Introduction

Visual impairment affects approximately 2.2 billion individuals worldwide, of which around 1 billion cases could have been prevented. The leading causes of this condition include uncorrected refractive errors and cataracts [1]. It is estimated that the prevalence of distance vision impairment is up to 4 times higher in low- and middle-income regions compared to high-income regions. The condition is more commonly observed in individuals aged 50 years and older [2].

Given this epidemiological context, visual impairment has a significant impact on multiple dimensions of an individual’s life, including physical, social, economic, and emotional aspects. Visual limitations compromise functional autonomy, hinder social interaction, restrict access to the labor market, and impose an economic burden on both the individual and public health systems [3,4]. This condition considerably affects quality of life, contributing to reduced productivity, increased dependency on others for daily activities, and, in many cases, early retirement [5,6]. Moreover, the emotional effects related to vision loss or limitation are often associated with feelings of isolation, depression, and decreased self-esteem [7-9]. In this context, assessing quality of life becomes essential for a comprehensive understanding of the consequences of visual impairment [10].

Quality of life is defined as an individual’s subjective perception of their position in life, situated within the cultural context and value systems in which they are embedded, and aligned with their personal goals, expectations, standards, and concerns [11]. This construct encompasses multiple domains, including physical, mental, and spiritual health; interpersonal relationships; educational level; work environment; socioeconomic status; sense of security; freedom; decision-making autonomy; social integration; and environmental conditions [12,13].

Accordingly, assessing quality of life is essential to identify the specific needs of individuals in particular contexts, enabling the development of targeted intervention strategies that address their demands and promote well-being and functionality [14,15].

A preliminary literature search conducted in February 2026 using the Web of Science and PubMed databases did not identify any review studies that specifically addressed this topic (Textbox 1). This finding highlights a gap in the scientific literature regarding the systematization of instruments used to assess quality of life in individuals with visual impairment.

Textbox 1. **Preliminary search.**

**Databases and search strategy**

- Web of Science: *((((Visual impairment) AND (Quality of life instruments)) OR (Quality of life assessment)) OR (Quality of life questionnaire)) (Topic) and Review Article (Document Types)*

- PubMed: *((((Visual impairment) AND (Quality of life instruments)) OR (Quality of life assessment)) OR (Quality of life questionnaire)) Filters: Review, Scoping Review, Systematic Review*

In this context, conducting a scoping review is justified as an appropriate methodological strategy to map and synthesize the available evidence on existing instruments used to assess the various domains of quality of life in this population. This approach is expected to provide valuable support for future research and clinical practice by guiding the selection of appropriate instruments available in the literature for measuring quality of life in individuals with visual impairment.

The study aims to map the available scientific evidence on the instruments used to assess the quality of life of individuals with visual impairment at any health care level.

## Methods

### Overview

A scoping review will be conducted following the methodological criteria established by the Joanna Briggs Institute [16] and guided by the PRISMA-ScR (Preferred Reporting Items for Systematic Reviews and Meta-Analyses Extension for Scoping Reviews) checklist [17]. The protocol was registered on the Open Science Framework (OSF) platform [18]. The population, concept, and context (PCC) framework was used to formulate the guiding research question: What instruments are available in the scientific literature to assess the quality of life of people with visual impairment across health care levels?

The PCC framework consisted of the following elements: P (population)—people with visual impairment, C (concept)—quality of life instruments, and C (context)—health care levels.

### Population

Studies related to individuals with visual impairment of any degree, caused by any etiology, whether unilateral or bilateral, will be included. All age groups will be included. The *International Classification of Diseases, 11th Revision* (*ICD-11*) defines visual impairment across different levels. Low vision is characterized by a visual acuity worse than 6/18 but equal to or better than 3/60, or by a visual field loss of less than 20° in the better eye with best possible correction. In contrast, blindness corresponds to a visual acuity worse than 3/60, or a visual field loss of less than 10° in the better eye, also with the best available correction [19].

### Concept

Studies addressing quality of life instruments will be included. Quality of life can be conceptualized from different perspectives; in this regard, one of the most widely accepted international definitions describes it as an individual’s perception of their position in life, considering the cultural context and value systems in which they are embedded, as well as their goals, expectations, and concerns [11]. It is therefore a multidimensional construct, which may encompass physical, psychological, social, and other dimensions [20,21]. Owing to this complexity, several instruments have been developed for its assessment, which may be generic in nature or targeted to specific health conditions and particular populations [22,23].

### Context

Studies conducted at any level of health care will be included, including primary, secondary, and tertiary care, as well as those addressing rehabilitation services and domiciliary care as shown in Textbox 2.

Textbox 2. **Inclusion and exclusion criteria.**

**Inclusion criteria**

- Articles derived from studies using various methodologies, such as experimental and quasi-experimental designs, analytical observational studies, descriptive observational studies, qualitative research, and systematic reviews
- Guidelines from professional societies, manuals, official protocols, government documents, theses, and dissertations
- Sources freely available in full text that report instruments used to assess quality of life in individuals with visual impairment
- Any publication year
- Any language of publication

**Exclusion criteria**

- Studies that do not address the research question
- Expert opinion pieces
- Incomplete studies
- Video or audio files will be excluded from the review

### Search Strategy

Searches will be conducted through the proxy access provided by the Federal University of Mato Grosso do Sul, via the Coordination for the Improvement of Higher Education Personnel (CAPES) Journal Portal, which provides extensive access to national and international bibliographic resources. This strategy allows the search to be broadened by including materials that are not available via open-access sources [24]. The following electronic databases will be searched: MEDLINE/PubMed (via the National Library of Medicine), Web of Science Core Collection (Clarivate Analytics), Embase (Elsevier), and Scopus (Elsevier). For gray literature, the following sources will be included: CAPES Theses and Dissertations Catalog, Google Scholar, Open Access Theses and Dissertations, and the World Health Organization website. In the CAPES catalog, the search strategy will use the terms *(deficiência visual) AND (avaliação da qualidade de vida)*. For the remaining sources, the terms *(visual impairment) AND (quality of life instruments) AND (quality of life assessment) AND (health service)* will be applied.

Additionally, reference lists of the included articles will be hand-searched to identify potentially relevant studies not captured in the database search, thereby reducing selection bias.

### Preliminary Search

An initial limited search was conducted in the Web of Science and PubMed databases to identify existing literature and refine the search terms (Table 1).

**Table 1.** Preliminary search results conducted on February 6, 2026.

Databases	Search query	Results, n
Web of Science	1 AND 2 AND 3	4515
PubMed	1 AND 2 AND 3	11,286

Descriptors were defined using the MeSH (Medical Subject Headings) and Emtree thesaurus. Based on this process search strategies were developed to retrieve studies related to each topic based on the protocol’s PCC framework:

1. Persons with visual impairment: *((((((Visual) OR (Visual disability)) OR (Visual impairment)) OR (Persons with visual impairment)) OR (Persons with visual disabilities)) OR (Visually impaired person)) OR (Visually Disabled Persons)*
2. Quality of life instruments: *((((((Quality of life instruments) OR (Quality of life assessment)) OR (Quality of life scale)) OR (Quality of life measure)) OR (Quality of life questionnaire)) OR (Quality of life evaluation)))*
3. Health care levels: *((((((((((((Health services) OR (Delivery of Health Care)) OR (Home Care Services)) OR (Rehabilitation)) OR (Primary Health Care)) OR (Primary Healthcare)) OR (Primary Care)) OR (Secondary Health Care)) OR (Secondary Healthcare)) OR (Secondary Care)) OR (Tertiary Health Care)) OR (Tertiary Healthcare)) OR (Tertiary Care)*

These preliminary findings demonstrate the relevance and variability of results based on search term combinations, highlighting the importance of applying comprehensive and sensitive strategies. To manage these numbers, the search strategy will be refined during the screening phase.

### Data Management

Duplicate records will be identified and removed to ensure that each reference is considered only once. Reference management will be conducted using Mendeley (Elsevier). Title and abstract screening will be performed through the Qatar Computing Research Institute platform (Rayyan QCRI) [25]. Data extraction and synthesis will be carried out using Microsoft Excel.

### Selection Process

The selection will occur in 3 stages. The first stage involves screening studies retrieved from the databases and exporting them in “RIS” format to Mendeley software for duplicate removal, followed by import into Rayyan QCRI for title and abstract screening.

For the second eligibility phase, reviewers will undergo training through a pilot test. After this, 2 or more independent, blinded reviewers will assess titles and abstracts against the review’s inclusion criteria using the Rayyan QCRI platform [25]. In cases of disagreement, a third researcher will evaluate the study to reach consensus.

The final stage consists of full-text reading of the included studies to verify compliance with the predefined inclusion criteria. The search results and study selection process will be fully reported in the final scoping review and illustrated with a PRISMA-ScR flow diagram [17].

### Data Extraction

Data synthesis will be conducted using Microsoft Excel spreadsheets. The variables to be extracted include: citation (authors and title), language, year of publication, publication medium (journal, guideline, thesis, etc), research location (country or region), objectives, study design, instrument name, validation status, type of instrument (questionnaires, interview guides, or tests), quality-of-life domains included in the instrument, target population for the instrument and application guidelines, mode of administration (self-administered or interviewer-administered), linguistic validations, and limitations.

### Presentation and Data Synthesis

Initially, the discussion will be structured based on categories or degrees of visual impairment, with people grouped according to these classifications. The data will be organized into tables, figures, and synoptic charts. A narrative summary will accompany these representations, highlighting the main findings and their relevance to the review’s objective.

## Results

**Figure 1.**
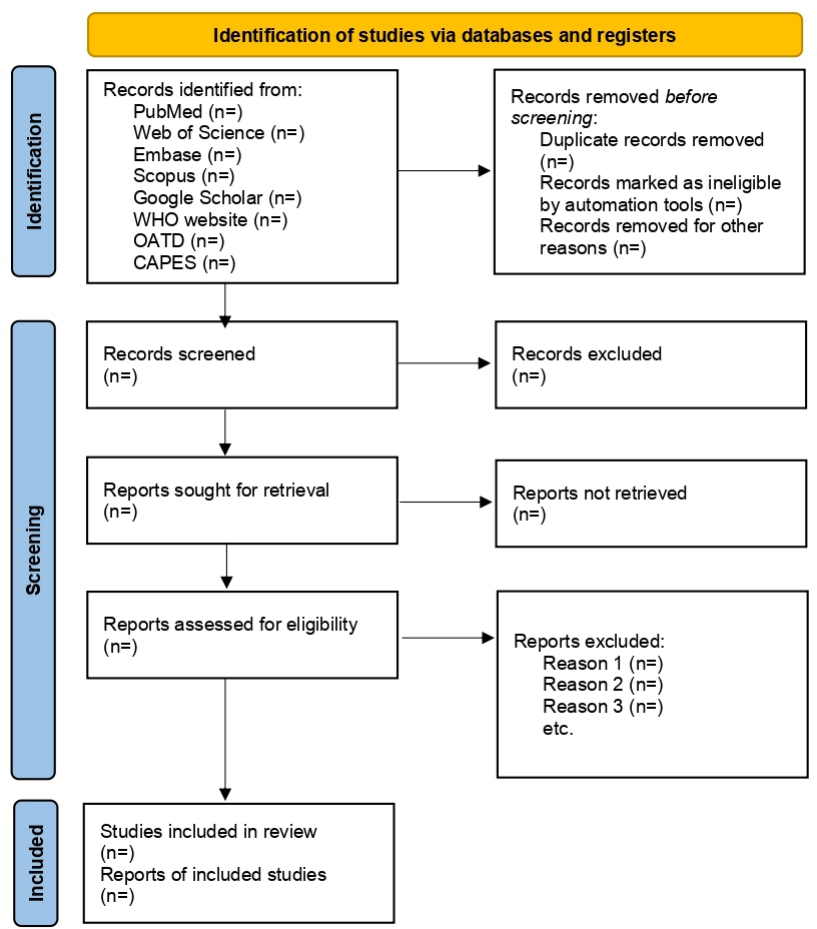
PRISMA (Preferred Reporting Items for Systematic Reviews and Meta-Analyses) 2020 flow diagram. CAPES: Coordination for the Improvement of Higher Education Personnel; WHO: World Health Organization.

This protocol was registered on the OSF Registries on July 28, 2025 [18]. Data collection is scheduled to take place from January to December 2026. The study selection process will be detailed using the PRISMA-ScR flow diagram (Figure 1). The results of this study will be disseminated through publication in a peer-reviewed scientific journal. It is expected that the findings will provide valuable support for the development and advancement of a broader research project.

## Discussion

### Anticipated Findings

This study protocol defines a rigorous approach to the design and implementation of a scoping review aimed at mapping the instruments used to assess the quality of life of individuals with visual impairments. The mapping of the studies will provide a comprehensive identification of the instruments used to assess the quality of life of individuals with visual impairment across different levels of health care. The synthesis of the data will enable the examination of relevant aspects, such as the development process of the instruments, the sociodemographic characteristics of the studied populations, and the quality of life domains (physical, social, and emotional) most frequently addressed. Furthermore, the identification of validation methods, modes of administration (self-administered or interviewer-administered), and the adaptations or translations conducted for different cultural contexts will help to understand where and how these tools have been most widely applied, as well as their applicability and methodological robustness, based on evidence extracted from studies that used instruments to assess quality of life [26-30].

The findings are expected to highlight the reliability and scientific basis of the available instruments, providing valuable support for the advancement of knowledge and professional practice in this field. Potential adjustments to this section may be necessary depending on the development and findings of the study.

### Future Directions

The study aims to address a critical gap in the scientific literature, as preliminary searches did not identify reviews that systematically compiled instruments specifically designed for this population. In the future, the findings of this review may contribute to strengthening public health policies aimed at promoting equity and inclusion, as well as guiding professional practice by assisting clinicians in selecting methodologically robust tools to assess the consequences of visual impairment on individuals’ lives. Additionally, the review may support further research by serving as a guide for researchers in the selection of appropriate instruments and in identifying areas that still lack specific assessment tools. Furthermore, it may contribute to improving living conditions by enabling more targeted interventions based on the real needs (physical, mental, and social) identified through these instruments.

### Limitations

Some limitations may arise during the course of the research. Among them, restricted access to articles, documents, and files that require payment may limit the scope of the analysis. Additionally, the selection of descriptors, as well as the choice of databases and sources of gray literature, may represent potential constraints that could lead to the exclusion of studies relevant to answering the research question.

### Conclusions

Therefore, it is essential to map, within the scientific literature, the instruments used to assess the quality of life of individuals with visual impairment. In this context, a scoping review enables the identification and synthesis of the available evidence, examining not only the existence of these instruments but also their characteristics, modes of application, validation processes, and contexts of use. Understanding these aspects is fundamental to recognizing methodologically robust and scientifically grounded tools capable of consistently assessing the quality of life of this population. In addition to contributing to the advancement of scientific knowledge, this mapping may support professional practice and inform the development of public health policies by guiding actions aimed at promoting equity, inclusion, and improvements in the living conditions of people with visual impairment.

## Abbreviations

CAPES: Coordination for the Improvement of Higher Education Personnel
*ICD-11*: *International Classification of Diseases, 11th Revision*
MeSH: Medical Subject Headings
OSF: Open Science Framework
PCC: population, concept, and context
PRISMA-ScR: Preferred Reporting Items for Systematic Reviews and Meta-Analyses Extension for Scoping Reviews
